# Human Cytomegalovirus and Risk of Incident Cardiovascular Disease in United Kingdom Biobank

**DOI:** 10.1093/infdis/jiab364

**Published:** 2021-07-19

**Authors:** Elizabeth M Hamilton, Naomi E Allen, Alexander J Mentzer, Thomas J Littlejohns

**Affiliations:** 1 Nuffield Department of Population Health, University of Oxford, Oxford, United Kingdom; 2 The Wellcome Centre for Human Genetics, University of Oxford, Oxford, United Kingdom

**Keywords:** human cytomegalovirus, cardiovascular disease, ischemic heart disease, stroke, UK Biobank, longitudinal

## Abstract

**Background:**

Previous studies have yielded conflicting results on the association between human cytomegalovirus (HCMV) and cardiovascular disease (CVD). This study examined associations between HCMV and incident CVD, ischaemic heart disease (IHD) and stroke.

**Methods:**

This study included 8531 women and men of predominantly white ethnic background, aged 40–69 without prevalent CVD from the population-based UK Biobank study, recruited between 2006–2010 with HCMV antibody levels measured. CVD was ascertained via linkage to health administrative records collected until 2020. Multivariate Cox proportional-hazards models were used to determine associations between HCMV seropositivity and incident CVD, IHD and stroke. HCMV seropositive antibody levels in tertiles were used to assess dose-response associations.

**Results:**

Over a mean follow-up period of 10.2 years, HCMV seropositivity was not significantly associated with CVD (Cases = 626, Hazard Ratio [HR] =1.01, 95% confidence interval [CI], .86–1.20), IHD (Cases = 539, HR=1.03, 95% CI, .87–1.24) or stroke (Cases = 144, HR = 0.96, 95% CI, .68–1.36). There was no evidence of dose-response associations with any outcome.

**Conclusions:**

We found no significant association between HCMV seropositivity and risk of CVD, IHD or stroke. Further research within understudied populations, such as those of non-white ethnicity, and CVD subtypes is warranted.

## Teaser

In this large cohort study of 8500 middle- to early older-aged, predominantly white, UK women and men we found no significant association between human cytomegalovirus past exposure and risk of incident cardiovascular disease, ischemic heart disease, or stroke.

Human cytomegalovirus (HCMV) is a common herpes virus transmitted by close contact, perinatal exposure, and blood transfusion with a seroprevalence between 60% and 85% in the United Kingdom [1]. Whilst HCMV increases morbidity and mortality risk in immunosuppressed individuals, in immunocompetent individuals it typically manifests as an asymptomatic lifelong latent infection that undergoes periodic subclinical reactivation. However, there is evidence that HCMV could be linked with an increased risk of cardiovascular disease (CVD) in the general population. Mechanistic research suggests that this could be driven through several pathways, including chronic inflammation, dysregulated vascular function, and repeat acute inflammatory reactions due to periodic subclinical reactivation, leading to accelerated atherogenesis [2]. Given CVD is the leading cause of death and disability globally [3], and HCMV is widely prevalent, yet not routinely screened for or treated, HCMV could potentially offer a promising target for CVD prevention.

However, the findings from observational studies using different CVD end points are mixed. In a meta-analysis of 10 prospective studies, HCMV was associated with a small increased risk of CVD, but was not significantly associated with ischemic heart disease (IHD) or stroke in subtype analyses [4]. Uncertainty remains due to the limitations of previous prospective studies, including small sample sizes (fewer than approximately 1500 participants) [5–12], populations with a high proportion of prevalent baseline comorbidities including CVD [5, 7–11], short follow-up periods (≤ 5 years) [7, 13, 14], insufficiently granular end points, and limited control for confounding factors [7, 9, 10]. Only one large (n = approximately 12 500) past prospective study has investigated dose-response associations between HCMV antibody levels and risk of IHD, finding evidence of a significant dose response [15]. Further investigation into potential dose-response effects based on titer levels are warranted.

To address these limitations in the research literature, we investigated the association between HCMV and incident CVD, including the subtypes IHD and stroke, in a large population-based prospective cohort study of middle-aged, largely healthy adults. We further investigated for dose-response associations using antibody titer levels.

## METHODS

### Population

Participants were selected from UK Biobank, a population-based prospective cohort study that recruited approximately half a million women and men aged 40–69 years between 2006 and 2010 [16]. All participants attended 1 of 22 baseline assessment centers located throughout England, Scotland, and Wales where they provided information on sociodemographic and lifestyle factors via a touchscreen questionnaire and verbal interview, underwent a range of physical measurements and provided blood samples. A subset of 20 000 participants attended a repeat of the baseline assessment during 2012 to 2013. UK Biobank received ethics approval from the National Information Governance Board for Health and Social Care and the National Health Service North West Multicentre Research Ethics Committee.

### HCMV Measurement

In 2016, a multiplex serology panel measuring immunoglobulin G (IgG) antibody responses to antigens of 20 infectious agents was performed on baseline serum samples of 9724 participants selected at random [17, 18]. Of these, 277 participants also had the panel performed on samples collected at repeat assessment. The panel included 3 HCMV antigens (pp28, pp52, and pp150 N-terminus). Samples were classified as seropositive if antibody responses to 2 out of the 3 HCMV antigens reached defined seroreactive median fluorescence intensity (MFI) thresholds, as described elsewhere [19].

### Assessment of Cardiovascular Outcomes

CVD was defined as a diagnosis of IHD or stroke and was ascertained using hospital inpatient records obtained from Hospital Episode Statistics for England, Scottish Morbidity Record for Scotland, and Patient Episode Database for Wales, as well as death registry records obtained from National Health Service Digital for England and Wales and Information and Statistics Division for Scotland. Diagnoses were recorded using the International Classification of Diseases (ICD)-9 coding system prior to 1996 and ICD-10 from 1996 onward. Primary or secondary hospital inpatient diagnoses and underlying causes of death were used to identify participants with CVD (see Supplementary Table 1 for list of ICD codes).

### Covariates

Townsend deprivation score was used as a measure of socioeconomic status and was assigned to participants based on their residential postcode at recruitment [20]. Ethnicity, education, smoking status, alcohol consumption, diabetes, hypertension, hypercholesterolemia, aspirin use, antihypertensive use, or cholesterol-lowering medication use were collected during the touchscreen questionnaire and verbal interview. Physical activity was assessed as metabolic equivalent of task (MET) hours per week, categorized into low, moderate, and high, derived in accordance with the International Physical Activity Questionnaire guidelines [21]. Body mass index (BMI; weight [kg]/height [m^2^]), and systolic and diastolic blood pressure (mmHg) were measured during the physical examination. C-reactive protein (CRP; mg/L), total cholesterol (mmol/L), low-density lipoprotein (LDL; mmol/L) cholesterol, high-density lipoprotein (mmol/L), and triglycerides (mmol/L) were measured from blood samples using clinical chemistry analyzers designed according to internationally recognized standards, and involved rigorous internal and external quality control procedures [22].

### Statistical Analysis

The distribution of baseline characteristics by HCMV serostatus were examined. Repeat assessment data were used to assess the repeatability of HCMV serostatus and to examine changes in HCMV antibody levels over time. For HCMV serostatus, Cohen κ statistic was calculated from participants with both baseline and repeat blood samples available.

Cox proportional-hazards regression models were used to estimate hazard ratios (HRs) of developing incident CVD in relation to HCMV seropositivity. Person-years were calculated from the date of recruitment until the date of first CVD diagnosis, date of death, date of loss to follow-up, or last date of hospital admission for England (31 March 2020), Scotland (31 October 2016), and Wales (29 February 2016), whichever came first. Separate models were performed for IHD and stroke as outcomes. Follow-up time, measured as person-years at risk, was used as the underlying time variable. There was no evidence of significant violation of the proportional-hazards assumption assessed by examining log-log plots.

Minimally adjusted models controlled for age (in years) and sex. Fully adjusted models included additional control for ethnicity (white, nonwhite), Townsend deprivation score (tertiles), education level (higher education [university or professional qualifications]; secondary school [A or O-level equivalent]; no education), smoking status (never; previous; current), alcohol intake (frequent [weekly or daily drinkers]; occasional [drinkers of other frequencies]; never drinkers), physical activity level (low; moderate; high), systolic blood pressure (in mmHg), BMI (in kg/m^2^), diabetes (no, yes), aspirin use (no, yes), antihypertensive use (no, yes), cholesterol-lowering medication use (no, yes), LDL (in mmol/L), and triglyceride levels (in mmol/L). For categorical variables, missing data, don’t know, or prefer not to answer responses (≤1.1%) were included in a separate category for analysis. Complete case analysis was used for continuous variables, as the proportion of missing values was low (≤2.4%).

In sensitivity analyses, the main models were repeated with exclusion for participants censored in the first 2 years of follow-up. This is to account for the possibility that participants might have underlying CVD at baseline, which results in hospitalization or death within short-term follow-up. The role of CRP as a mediator, modifier, or confounder in the HCMV-CVD association is controversial [4, 8, 23], and therefore was not included in the fully adjusted multivariable model, but rather examined in sensitivity analyses as an additional level of adjustment, and effect modification by CRP tertiles examined by addition of interaction terms with HCMV serostatus.

To investigate the dose-response association between HCMV antibody levels and total CVD, IHD, and stroke, analyses were performed on natural log-transformed HCMV pp28 antibody titer levels (hereon referred to as HCMV antibodies). This antibody was selected because it had the highest specificity (99%) and sensitivity (97%) for detecting HCMV serostatus compared to the gold standard assay during validation [19]. The pp28 seroreactive threshold was defined by the trough level between the distinct natural log-transformed bimodal normal distributions, corresponding to a natural log-transformed MFI value of 6.0. Seropositive participants were categorized into tertiles based on natural log-transformed MFI values (low, 6.0–7.3; medium, 7.4–7.9; high, 8.0–8.3), and compared to seronegative participants (<6.0). Repeat measures taken 4 to 5 years later were used to calculate regression dilution ratios using the MacMahon-Peto method [24]. Linear tests of trend across seropositive tertiles as an ordinal variable were performed to assess for the presence of a dose-response association. Statistical analysis was performed in Stata (version 14.2) and statistical significance was defined at *P* < .05.

## RESULTS

**Figure 1.**
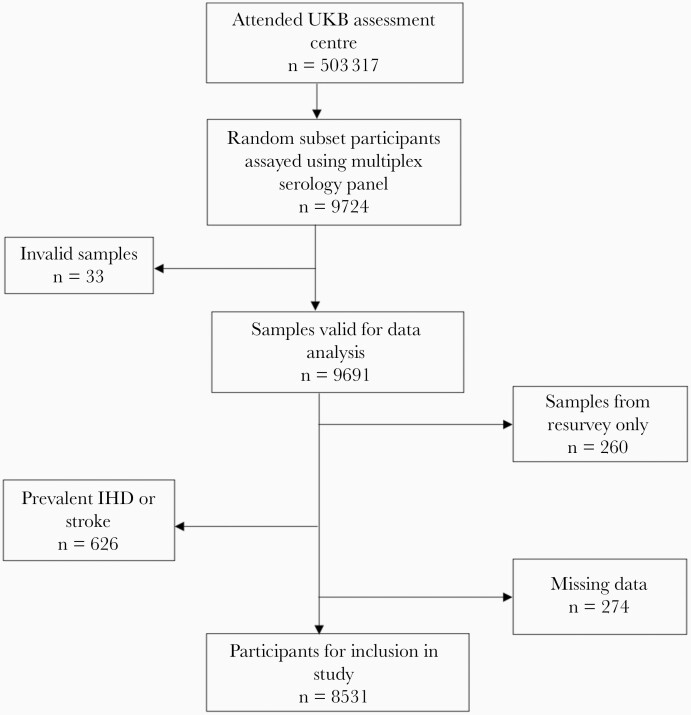
Flow diagram showing participant selection for inclusion in the study. Abbreviations: IHD, ischemic heart disease; UKB, UK Biobank.

Of the 9691 participants who had valid HCMV serostatus data, 626 participants with prevalent CVD, 260 participants with only resurvey data, and 274 participants with missing data were excluded, resulting in a final sample of 8531 participants (Figure 1). For secondary analyses there were 8631 and 9010 participants for IHD and stroke analyses, respectively. Of the 626 incident CVD events over a mean follow-up time of 10.2 years (SD = 2.1 years), 599 were nonfatal events and 27 were fatal events. For IHD and stroke separately, there were 539 incident IHD events, of which 516 were nonfatal and 23 were fatal, and 144 incident strokes, of which 136 were nonfatal and 8 were fatal.

Of the 8531 participants, 57.9% (n = 4938) were seropositive at baseline. Of 255 included participants with measurements at repeat assessment, there was a seroconversion rate of 10% (n = 13) among participants who were seronegative at baseline, and a seroreversion rate of 4% (n = 5) among participants who were seropositive at baseline. This generated a Cohen κ statistic of 0.86, indicating a substantial level of agreement between baseline and repeat serostatus measured a mean of 4.5 years (SD = 0.8 years) apart. For HCMV antibody levels, the regression dilution ratio was high at 0.87 (Supplementary Table 2), indicating resurvey antibody measurements were very highly correlated with baseline measurements, and thus findings were not corrected for regression dilution bias (see Supplementary Table 3 for characteristics of participants at repeat assessment).

Overall, compared with seronegative participants, those who were seropositive were more likely to be older, female, of nonwhite ethnic origin, live in an area of higher socioeconomic deprivation, have fewer qualifications, have a higher BMI, higher blood pressure, and were more likely to have diabetes, hypertension, and hypercholesterolemia (Table 1). The distribution of smoking, physical activity, total cholesterol, LDL, and triglyceride levels did not vary materially by HCMV serostatus, while frequent alcohol drinking was more common in seronegative participants.

**Table 1.** Baseline Characteristics of 8531 UK Biobank Study Participants by HCMV Serostatus

Characteristics	Overall Population (n = 8531)	HCMV Seronegative (n = 3593, 42.1%)	HCMV Seropositive (n = 4938, 57.9%)
Age, y, mean (SD)	56.2 (8.2)	54.9 (8.3)	57.0 (7.9)
Age group, y			
40–49	2137 (25.0)	1111 (30.9)	1026 (20.8)
50–59	2840 (33.3)	1191 (33.1)	1649 (33.4)
60–70	3554 (41.7)	1291 (35.9)	2263 (45.8)
Sex			
Male	3630 (42.6)	1596 (44.4)	2034 (41.2)
Female	4901 (57.4)	1997 (55.6)	2904 (58.8)
Ethnic background			
White	8039 (94.2)	3533 (98.3)	4506 (91.3)
Nonwhite	451 (5.3)	48 (1.3)	403 (8.2)
Not reported/missing	41 (0.5)	12 (0.3)	29 (0.6)
Deprivation			
Least deprived third	4542 (53.2)	2000 (55.7)	2542 (51.5)
Middle third	2690 (31.5)	1125 (31.3)	1565 (31.7)
Most deprived third	1291 (15.1)	465 (12.9)	826 (16.7)
Not reported/missing	8 (0.1)	3 (0.1)	5 (0.1)
Education level			
Higher education	5103 (59.8)	2273 (63.3)	2830 (57.3)
Secondary school	1986 (23.3)	866 (24.1)	1120 (22.7)
No education	1350 (15.8)	429 (11.9)	921 (18.7)
Not reported/missing	92 (1.1)	25 (0.7)	67 (1.4)
Smoking status			
Never smoker	4819 (56.5)	2103 (58.5)	2716 (55.0)
Exsmoker	2836 (33.2)	1127 (31.4)	1709 (34.6)
Current smoker	827 (9.7)	351 (9.8)	476 (9.6)
Not reported/missing	49 (0.6)	12 (0.3)	37 (0.7)
Alcohol intake			
Never	1962 (23.0)	779 (21.7)	1183 (24.0)
Occasional	5919 (69.4)	2584 (71.9)	3335 (67.5)
Frequent	634 (7.4)	226 (6.3)	408 (8.3)
Not reported/missing	16 (0.2)	4 (0.1)	12 (0.2)
Physical activity level			
Low	1313 (15.4)	556 (15.5)	757 (15.3)
Moderate	2746 (32.2)	1210 (33.7)	1536 (31.1)
High	2829 (33.2)	1186 (33.0)	1643 (33.3)
Not reported/missing	1643 (19.3)	641 (17.8)	1002 (20.3)
Systolic blood pressure, mmHg, mean (SD)	139.7 (19.9)	138.6 (19.6)	140.5 (20.1)
Diastolic blood pressure, mmHg, mean (SD)	82.4 (10.7)	82.1 (10.7)	82.6 (10.8)
Body mass index, kg/m^2^, mean (SD)	27.2 (4.7)	26.8 (4.6)	27.4 (4.8)
Medical conditions			
Diabetes	337 (4.0)	120 (3.3)	217 (4.4)
Hypertension	2014 (23.6)	793 (22.1)	1221 (24.7)
Hypercholesterolemia	1178 (13.8)	436 (12.1)	742 (15.0)
Medication use			
Aspirin	801 (9.4)	299 (8.3)	502 (10.2)
Antihypertensive	751 (8.8)	277 (7.7)	474 (9.6)
Cholesterol lowering	1081 (12.7)	391 (10.9)	690 (14.0)
Biomarkers			
Total cholesterol, mmol/L, mean (SD)	5.8 (1.1)	5.8 (1.1)	5.8 (1.1)
LDL cholesterol, mmol/L, mean (SD)	3.6 (0.9)	3.6 (0.9)	3.6 (0.9)
Triglycerides, mmol/L, mean (SD)	1.7 (1.0)	1.7 (1.0)	1.7 (1.0)
C-reactive protein, mg/L, median (IQR)	1.3 (0.7–2.7)	1.2 (0.6–2.6)	1.4 (0.7–2.8)

Data are No. (%) unless otherwise indicated.

Abbreviations: HCMV, human cytomegalovirus; IQR, interquartile range; LDL, low-density lipoprotein.

**Figure 2.**
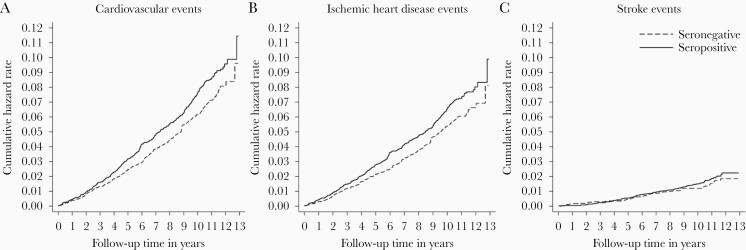
*A*–*C*, Nelson-Aalen curves of cumulative hazard of cardiovascular events by human cytomegalovirus status.

In the main analyses, although slight differences in the cumulative hazard rate of CVD events emerged between HCMV-seropositive and -seronegative participants (unadjusted HR = 1.20; 95% confidence interval [CI], 1.02–1.41; Figure 2 and Table 2), after adjustment for age and sex, HCMV seropositivity was not significantly associated with an increased risk of CVD (HR = 1.09; 95% CI, .93–1.28; Table 2). The association was further attenuated after additional adjustment for a range of sociodemographic, lifestyle, and health-related characteristics (HR = 1.01; 95% CI, .86–1.20). Similar patterns of association were observed for IHD (unadjusted HR = 1.21 [95% CI, 1.02–1.44]; minimally adjusted HR = 1.11 [95% CI, .93–1.33]; fully adjusted HR = 1.03 [95% CI, .87–1.24]) and stroke (unadjusted HR = 1.20 [95% CI, .85–1.68]; minimally adjusted HR = 1.01 [95% CI, .72–1.41]; fully adjusted HR = 0.96 [95% CI, .68–1.36]; Table 2).

**Table 2.** Association Between HCMV Serostatus and Incident Cardiovascular Disease

Outcome	Total No. of Participants	No. of Events	Unadjusted Model, HR (95% CI)	Minimally Adjusted Model,^a^ HR (95% CI)	Fully Adjusted Model,^b^ HR (95% CI)
Cardiovascular disease	8531	626	1.20 (1.02–1.41)	1.09 (.93–1.28)	1.01 (.86–1.20)
Ischemic heart disease	8631	539	1.21 (1.02–1.44)	1.11 (.93–1.33)	1.03 (.87–1.24)
Stroke	9010	144	1.20 (.85–1.68)	1.01 (.72–1.41)	0.96 (.68–1.36)

Abbreviations: CI, confidence interval; HCMV, human cytomegalovirus; HR, hazard ratio.

^a^Adjusted for age and sex.

^b^Adjusted for age, sex, ethnicity, socioeconomic deprivation, education, smoking, alcohol, physical activity, systolic blood pressure, body mass index, diabetes, aspirin, cholesterol-lowering or antihypertensive use, LDL, and triglycerides.

In sensitivity analyses that excluded participants censored within 2 years of blood collection (n = 121), the fully adjusted multivariate HRs were 1.02 (95% CI, .86–1.22), 1.03 (95% CI, .85–1.24), and 1.09 (95% CI, .75–1.59) for total CVD, IHD, and stroke, respectively.

**Figure 3.**
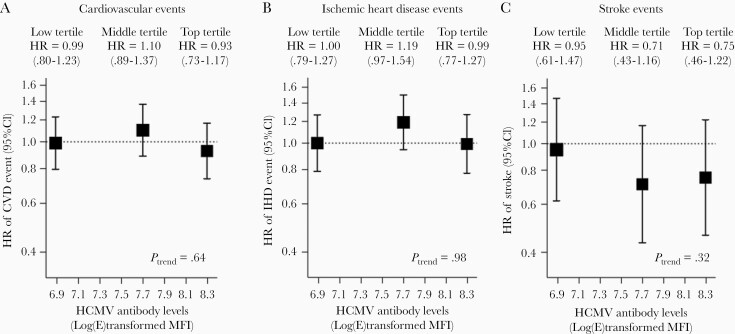
*A*–*C*, Association between HCMV-seropositive antibody tertiles compared to seronegative participants and incident cardiovascular disease. HR (95% CI) for seropositive tertiles are presented. Antibody group log(E)transformed MFI ranges: low antibody group, 6–7.3; middle antibody group, 7.4–7.9; high antibody group 8.0–8.3. Models adjusted for age, sex, ethnicity, socioeconomic deprivation, education, smoking, alcohol, physical activity, systolic blood pressure, body mass index, diabetes, aspirin, cholesterol-lowering, or antihypertensive use, low-density lipoprotein, and triglycerides. Abbreviations: CI, confidence interval; HCMV, human cytomegalovirus; HR, hazard ratio; MFI, median fluorescence intensity.

In analyses that investigated seropositive antibody titer levels in association with each outcome, the fully adjusted HRs of total CVD for participants in low, middle, and high tertiles of HCMV seropositivity were 0.99 (95% CI, .80–1.23), 1.10 (95% CI, .89–1.37), and 0.93 (95% CI, .73–1.17), respectively (*P* for trend = .64; Figure 3A) compared to seronegative participants. Similarly, there was also no evidence of a dose-response relationship between HCMV and IHD (*P* for trend = .98) or stroke (*P* for trend = .32) (Figure 3B and 3C). Sensitivity analyses additionally adjusted for CRP were not significant (Supplementary Table 4) and there was no evidence of significant effect modification of the HCMV-CVD association by CRP tertiles (*P* = .23; Supplementary Figure 1).

## Discussion

In this large, population-based prospective cohort of approximately 8500 middle- to older-aged, predominately white, UK-based women and men without prevalent CVD, we did not find evidence that past infection with HCMV was significantly associated with an increased risk of CVD, IHD, or stroke. We found that age, sex, and other traditional cardiovascular risk factors were strong confounders of the HCMV-CVD association.

Our findings overlap with studies included in a recent systematic review performed by Wang et al [4]. In their meta-analysis of 10 prospective cohort studies, they reported that HCMV seropositivity was associated with a 22% (relative risk [RR] = 1.22; 95% CI, 1.07–1.38) increased risk of CVD compared to seronegative individuals, including studies with various levels of adjustment for confounding. However, in subtype analyses, this statistically significant association only remained for cardiovascular mortality (RR = 1.30; 95% CI, 1.03–1.66), whilst the pooled effect estimates for IHD (RR = 1.16; 95% CI, .95–1.42) and stroke (RR = 1.16; 95% CI, .67–2.01) were attenuated. Furthermore, selection bias could have biased these estimates: of the 10 studies included, 2 were performed in participants with known baseline CVD [5, 25], 1 was conducted in patients referred to a cardiovascular prevention unit [7], and a further 3 [8–10] were in elderly patients (with a mean age between 70 to 85 years) and those with baseline CVD were not excluded. Furthermore, 2 of the studies [9, 10] that reported significant positive associations between HCMV and cardiovascular mortality only adjusted for sex and/or age and thus could have been affected by confounding factors. In the current study, we found that adjustment for potential confounders, in addition to age and sex, further attenuated the observed associations. The largest cohort study included was that performed by Simanek et al [23], which used the National Health and Nutritional Examination Survey III cohort (NHANES) of 14 000 middle-aged Americans followed up for an average of 13.7 years. They reported an HR of 1.19 (95% CI, .95–1.49) of cardiovascular mortality in seropositive compared to seronegative participants; however, they did not report excluding participants with prevalent CVD. We were unable to explore the association with CVD mortality in the current study due to a lack of CVD deaths.

The nonsignificant association between HCMV seropositivity and IHD in our study is consistent with 9 prospective studies identified in the literature. Four studies [12, 15, 26, 27] reported that HCMV seropositivity was associated with a nonsignificant increase in IHD, and 5 studies [5, 28–31] reported a nonsignificant decrease in IHD. Regarding stroke, our finding of a null association overlaps with a 2018 systematic review and meta-analysis of 12 observational studies that included 7424 participants with 1143 cases of stroke [32]. They reported overall effect estimates for HCMV and stroke of 1.40 (95% CI, .67–2.96) and 1.01 (95% CI, .73–1.39) for case-control and cohort studies respectively. However, the authors deemed that 10 of 12 included studies had high risk of bias in at least 1 domain, including 7 for inadequate adjustment for confounding and 6 for inability to determine temporality, where CMV was recorded after stroke diagnosis. Furthermore, the present study, along with most past studies, did not have the power to investigate stroke subtypes and more accurate stroke phenotyping should be performed.

We also investigated the shape of the HCMV and CVD association across antibody titer levels and did not find significant dose-response relationships in association with CVD, IHD, or stroke. Although confidence intervals across seropositive tertiles are wide and overlapping, these findings add to the limited extant evidence base around the association between HCMV antibody levels and CVD risk. Gkrania-Klotsas et al [15], reported that seropositive participants in the highest antibody tertile were at a 21% (95% CI, 4%–41%) increased risk of IHD compared to seronegative participants. While their study is comparable to ours in terms of demographics and health status of the study population, they had considerably more events (1356 IHD cases) and hence greater statistical power to detect an association. Roberts et al [8], in an elderly Latino cohort (n = approximately 1500), reported a nonsignificantly elevated risk of cardiovascular death in participants in the highest quartile of HCMV antibodies compared to the lower 3 quartiles combined. Two small case-control studies by Strachan et al [12] (n = approximately 400) and Sorlie et al [14] (n = approximately 700) described nonsignificant U-shaped associations across HCMV quartiles with IHD, and a combined CVD end point (MI, CHD death, or revascularization), respectively. For HCMV antibody levels and stroke, 2 prospective studies found a nonsignificant decrease in stroke risk in the higher antibody groups compared to lower groups [26, 33]. Much of this existing evidence is limited by small sample size, and therefore to elicit the true shape of any association, more power is needed. Furthermore, to enable meaningful comparison and synthesis of existing evidence in relation to dose-response associations between HCMV antibodies and CVD, use of standardized HCMV assays and methods for generating thresholds to categorize HCMV antibodies is needed.

The current study has several strengths. It combines a large sample size with detailed measurements to enable adjustment for a range of confounders and a long follow-up period. Aside from Gkrania-Klotsas et al [15] (n = approximately 12 500) and Simanek et al [23] (n = approximately 14 000, which reported only CVD death) previous studies have consisted of less than 2000 participants. The depth of data in UK Biobank enabled rigorous adjustment for a range of potential sources of confounding, although a degree of residual confounding likely remains. The multiplex serology panel has a high assay performance, with a specificity of 96.9% to 100% and sensitivity of 96% to 100% [18, 19]. The use of cohort-wide electronic health records to ascertain CVD outcomes ensures that loss to follow-up is minimal.

This study also has several limitations. Firstly, UK Biobank is not representative of the general population due to healthy volunteer selection bias and cannot be used to estimate seroprevalence rates [34]. Additionally, given the low percentage of participants of nonwhite ethnicity in UK Biobank, our findings may not be generalizable to nonwhite populations. As the prevalence of HCMV is known to be higher in nonwhite populations, further research in samples enriched for nonwhite participants is warranted. Secondly, hospital inpatient and death records will not capture all CVD events [35] (ie, some will only be identified through linkage to primary care records), although previous research has found that inpatient records have a high level of validity for CVD, IHD, and stroke [36]. Third, HCMV antibody levels used to investigate dose-response associations may be raised because of factors other than infection, including duration of infection, host immune status, and presence of coinfections [37], and may not necessarily be a reliable marker of HCMV infection burden. Fourth, the presence of coinfections was not assessed in this study. Future research should focus on using methods to directly detect HCMV, for example via detection of HCMV DNA load, and controlling for other putative infectious risk factors.

Finally, this study, although large in size compared to existing studies, may not have the power to reliably detect modest effects of HCMV on CVD. Given the magnitude of the public health burden associated with CVD worldwide it is important that such modest effects of HCMV on CVD are reliably described. Wang et al [4] in their meta-analysis of prospective studies on HCMV and CVD, using their pooled RR of 1.22 (95% CI, 1.07–1.38) and HCMV prevalence of 70.1%, estimated that 13.4% (95% CI, 12.0%–14.5%) of CVD events could be attributed to HCMV. This is larger than other well-established modifiable risk factors such as smoking, poor diet, low education, obesity, and diabetes, which were each reported to have a CVD population attributable fraction of 5% to 6% in a cohort of 155 722 people from 21 high-, middle-, and low-income countries [38]. However, whilst CMV might be a modifiable risk factor for CVD, we did not find evidence of a significant association, and the addition of our findings to previously published meta-analyses is likely to further attenuate the association observed for CVD, IHD, and stroke.

In summary, we did not find a significant association between past HCMV infection and subsequent risk of CVD, IHD, or stroke in healthy middle- to older-aged UK adults. Further research on CVD subtypes that have been under investigated such as stroke subtypes, and in more diverse populations (in particular those of nonwhite ethnicity) are warranted.

## Supplementary Data

Supplementary materials are available at *The Journal of Infectious Diseases* online. Consisting of data provided by the authors to benefit the reader, the posted materials are not copyedited and are the sole responsibility of the authors, so questions or comments should be addressed to the corresponding author.

jiab364_suppl_Supplementary_Data

## Notes

***Acknowledgments.*** The authors thank all UK Biobank study participants, UK Biobank research staff, UK Biobank steering committee, and colleagues at the Infections and Cancer Epidemiology, German Cancer Research Centre for their validation work on the UK Biobank multiplex serology panel. This research has been conducted using the UK Biobank Resource under application number 43920.

***Financial support.*** This work was supported by the National Institute for Health Research Biomedical Research Centre (grant supporting DPhil studies to E. M. H.). UK Biobank is supported by the Wellcome Trust, Medical Research Council, UK Department of Health, the Scottish Government, the Welsh Assembly Government, British Heart Foundation, Cancer Research UK, and Diabetes UK.

***Potential conflicts of interest.*** All authors: No reported conflicts of interest. All authors have submitted the ICMJE Form for Disclosure of Potential Conflicts of Interest. Conflicts that the editors consider relevant to the content of the manuscript have been disclosed.
