# Supplementary material for: Human Cytomegalovirus and Risk of Incident Cardiovascular Disease in United Kingdom Biobank
Source: J Infect Dis. 2021 Jul 19;225(7):1179–88. doi: 10.1093/infdis/jiab364 (PMC8974830; doi:10.1093/infdis/jiab364)
Supplement: jiab364_suppl_Supplementary_Data [file jiab364_suppl_supplementary_data.docx]

# Supplementary Data File

**Supplementary Table 1:** Cardiovascular disease endpoints and corresponding International Classification of Disease 9 and 10 codes.

| **Outcome** | **ICD-10 Endpoint** | **ICD-10 Code** | **ICD-9 Endpoint** | **ICD-9 Code** |
| --- | --- | --- | --- | --- |
| **Ischaemic heart disease** | Angina pectoris | I20 | Acute myocardial infarction | 410 |
|  | Acute myocardial infarction | I21 | Other acute and subacute forms of ischaemic heart disease | 411 |
|  | Subsequent myocardial infarction | I22 | Old myocardial infarction | 412 |
|  | Certain current complications following acute myocardial infarction | I23 | Angina pectoris | 413 |
|  | Other acute ischaemic heart diseases | I24 | Other forms of chronic ischaemic heart disease | 414 |
|  | Chronic ischaemic heart disease | I25 |  |  |
| **Stroke of any type*** | Subarachnoid haemorrhage | I60 | Subarachnoid haemorrhage | 430.X |
|  | Intracerebral haemorrhage | I61 | Intracerebral haemorrhage | 431.X |
|  | Ischaemic stroke | I63 | Occlusion of cerebral arteries | 434.X |
|  | Unspecified stroke | I64.X | Cerebral thrombosis | 434.0 |
|  |  |  | Cerebral embolism | 434.1 |
|  |  |  | Cerebral artery occlusion, unspecified | 434.9 |
|  |  |  | Acute, but ill-defined, cerebrovascular disease | 436.X |

Abbreviations: ICD, International Classification of Disease.

**Supplementary Table 2:** Calculation of the regression dilution ratio for HCMV antibodies using the MacMahon-Peto method

| Quintiles of baseline antibodies | N baseline samples (n=8,531) | Baseline mean antibody level  (log-transformed MFI) | N repeat samples (n=255) | Repeat mean antibody level  (log-transformed MFI) |
| --- | --- | --- | --- | --- |
| I | 1,714 | 3.5 | 80 | 4.0 |
| II | 1,702 | 4.7 | 44 | 4.4 |
| III | 1,705 | 6.1 | 46 | 6.1 |
| IV | 1,704 | 7.5 | 38 | 7.5 |
| V | 1,706 | 8.2 | 47 | 8.1 |
| Difference (V-1) |  | 4.7 |  | 4.1 |
| RDR |  |  |  | 0.87 |

Abbreviations: MFI, median fluorescence intensity; N, number; RDR, regression dilution ratio

**Supplementary Table 3:** Key baseline characteristics of 255 UK Biobank study participants by HCMV serostatus at repeat serosurvey

| **Mean (SD) or Number (%)** | **Overall population**  (n=255) | **HCMV Seronegative**  (n=132, 51.8%) | **HCMV Seropositive**  (n=123, 48.2%) |
| --- | --- | --- | --- |
| **Age (years),** mean (sd) | 56.5 (7.4) | 55.1 (8.1) | 58.0 (6.3) |
| **Sex** |  |  |  |
| Male | 105 (41.2) | 54 (40.9) | 51 (41.5) |
| Female | 150 (58.8) | 78 (59.1) | 72 (58.5) |
| **Ethnic background** |  |  |  |
| White | 245 (96.1) | 130 (98.5) | 115 (93.5) |
| Non-white | 7 (2.7) | 0 (0.0) | 7 (5.7) |
| Not reported | 3 (1.2) | 2 (1.5) | 1 (0.8) |
| **Deprivation** |  |  |  |
| Least deprived third | 154 (60.4) | 81 (61.4) | 73 (59.3) |
| Middle third | 76 (29.8) | 38 (28.8) | 38 (30.9) |
| Most deprived third | 25 (9.8) | 13 (9.8) | 12 (9.8) |
| **Education level** |  |  |  |
| Higher education | 172 (67.5) | 88 (66.7) | 84 (68.3) |
| Secondary school | 51 (20.0) | 31 (23.5) | 20 (16.3) |
| No education | 31 (12.2) | 13 (9.8) | 18 (14.6) |
| Not reported | 1 (0.4) | 0 (0) | 1 (0.8) |

Numbers (%) are presented, unless otherwise stated

Abbreviations: HCMV, human cytomegalovirus;

**Supplementary Table 4:** Association between HCMV serostatus and incident cardiovascular disease additionally adjusted for C-reactive protein

| **Outcome** | **Fully adjusted model^a^**  **(HR, 95% CI)** |
| --- | --- |
| **Cardiovascular disease** | 1.01 (0.86, 1.20) |
| **Ischaemic heart disease** | 1.03 (0.87, 1.24) |
| **Stroke** | 0.96 (0.68, 1.36) |

^a^Adjusted for age, sex, ethnicity, socioeconomic deprivation, education, smoking, alcohol, physical activity, systolic blood pressure, body mass index, diabetes, aspirin, cholesterol-lowering or antihypertensive use, LDL, triglycerides and CRP.

Abbreviations: HCMV, human cytomegalovirus, CRP, C-reactive protein.

**Supplementary Figure 1:** Cox proportional-hazards models for the association between HCMV serostatus and CVD stratified by CRP tertiles^a^


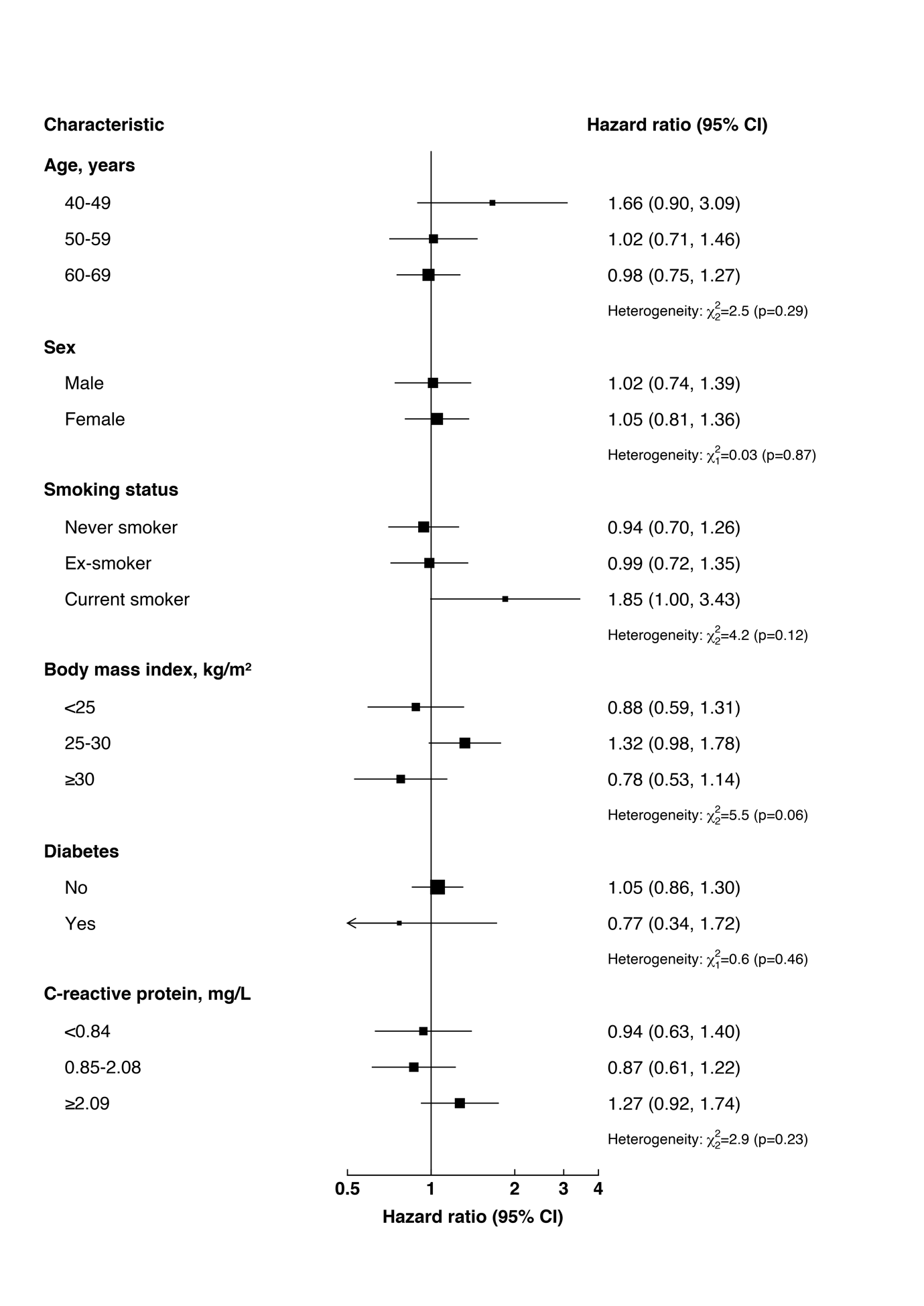

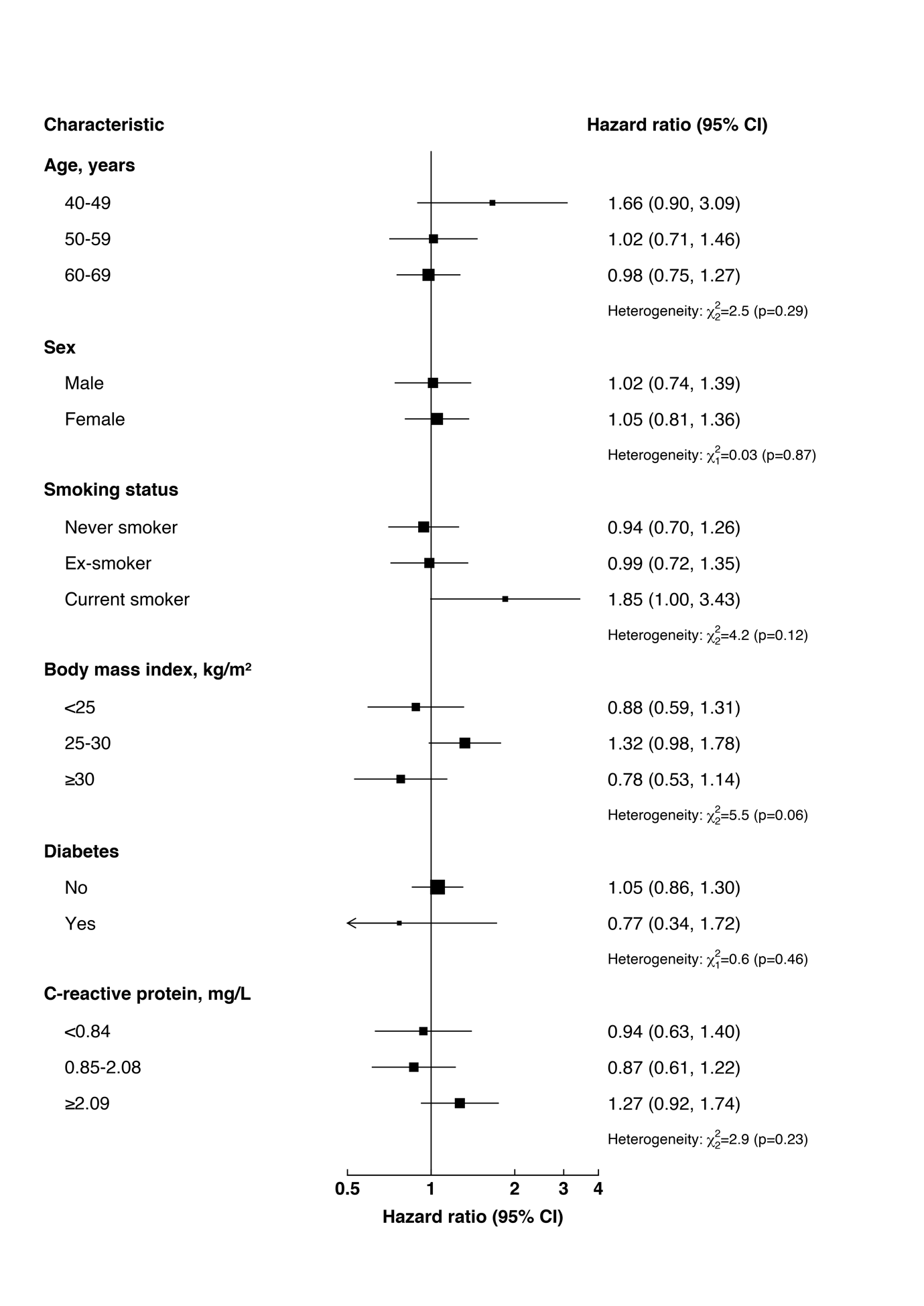


^a^Tertiles of CRP (mg/L): Bottom tertile <0.84; Middle tertile: 0.85-2.08; Top tertile: ≥ 2.09

Abbreviations: CI, confidence interval; HCMV, human cytomegalovirus
